# Establishing evidence criteria for implementation strategies in the US: a Delphi study for HIV services

**DOI:** 10.1186/s13012-024-01379-3

**Published:** 2024-07-15

**Authors:** Virginia R. McKay, Alithia Zamantakis, Ana Michaela Pachicano, James L. Merle, Morgan R. Purrier, McKenzie Swan, Dennis H. Li, Brian Mustanski, Justin D. Smith, Lisa R. Hirschhorn, Nanette Benbow

**Affiliations:** 1 Center for Public Health Systems Science, Brown School, Washington University in St. Louis, St. Louis, MO USA; 2 Department of Medical Social Sciences, Feinberg School of Medicine, Northwestern University, Chicago, IL USA; 3 Institute for Sexual and Gender Minority Health and Wellbeing, Northwestern University, Chicago, IL USA; 4 Department of Population Health Sciences, Division of Health System Innovation and Research, Spencer Fox Eccles School of Medicine, The University of Utah, Salt Lake City, UT USA; 5 Center for Prevention Implementation Methodology, Feinberg School of Medicine, Northwestern University, Chicago, IL USA; 6 Department of Psychiatry and Behavioral Sciences, Feinberg School of Medicine, Northwestern University, Chicago, IL USA; 7 Center for Dissemination and Implementation Science, Feinberg School of Medicine, Northwestern University, Chicago, IL USA

**Keywords:** HIV, Evidence-based intervention, Implementation science

## Abstract

**Background:**

There are no criteria specifically for evaluating the quality of implementation research and recommending implementation strategies likely to have impact to practitioners. We describe the development and application of the Best Practices Tool, a set of criteria to evaluate the evidence supporting HIV-specific implementation strategies.

**Methods:**

We developed the Best Practices Tool from 2022–2023 in three phases. (1) We developed a draft tool and criteria based on a literature review and key informant interviews. We purposively selected and recruited by email interview participants representing a mix of expertise in HIV service delivery, quality improvement, and implementation science. (2) The tool was then informed and revised through two e-Delphi rounds using a survey delivered online through Qualtrics. The first and second round Delphi surveys consisted of 71 and 52 open and close-ended questions, respectively, asking participants to evaluate, confirm, and make suggestions on different aspects of the rubric. After each survey round, data were analyzed and synthesized as appropriate; and the tool and criteria were revised. (3) We then applied the tool to a set of research studies assessing implementation strategies designed to promote the adoption and uptake of evidence-based HIV interventions to assess reliable application of the tool and criteria.

**Results:**

Our initial literature review yielded existing tools for evaluating intervention-level evidence. For a strategy-level tool, additions emerged from interviews, for example, a need to consider the context and specification of strategies. Revisions were made after both Delphi rounds resulting in the confirmation of five evaluation domains – research design, implementation outcomes, limitations and rigor, strategy specification, and equity – and four evidence levels – best, promising, more evidence needed, and harmful. For most domains, criteria were specified at each evidence level. After an initial pilot round to develop an application process and provide training, we achieved 98% reliability when applying the criteria to 18 implementation strategies.

**Conclusions:**

We developed a tool to evaluate the evidence supporting implementation strategies for HIV services. Although specific to HIV in the US, this tool is adaptable for evaluating strategies in other health areas.

**Supplementary Information:**

The online version contains supplementary material available at 10.1186/s13012-024-01379-3.

Contributions to the literature

- The field of implementation science has not yet established criteria to evaluate the quality of evidence for implementation strategies.
- Our Delphi process with experts in implementation science, quality improvement, and HIV services identified criteria that should be used to evaluate evidence for HIV-related implementation research in the US.
- Our tool can be applied to cases of HIV-related implementation research to make recommendations to practitioners implementation strategies most likely to be effective. They could also be adapted to other health domains.

## Introduction

Implementation science is dedicated to improving the uptake and use of evidence-based interventions, practices, and policies to capitalize on scientific knowledge and impact human health. Central to the goals of implementation research is building the evidence for implementation strategies, defined as techniques or change efforts to promote the adoption, implementation, and sustainment of evidence-based interventions (EBIs) [1]. In a recent review, scholars within the field of implementation science recognized that a more robust research agenda related to implementation strategies is needed to yield the promised benefits of improved EBI implementation for practitioners [2]. Within this agenda is a call for more research on the effectiveness of implementation strategies. Expanding on this priority, criteria on which to evaluate evidence quality are needed to assess whether the evidence supporting the effectiveness of any given strategy is sufficient. Without criteria on which to evaluate implementation research focusing on strategies, it is difficult to recommend strategies that are likely to be the most valuable for practitioners or to identify strategies that may hold initial promise but would benefit from more robust research. Evidence criteria are also an foundational element of the creation of a compendium of evidence-based implementation strategies, which is a key dissemination approach for delivering evidence to implementers.

At the intervention level, criteria and rubrics are available to synthesize research outcomes and evaluate research quality behind the evidence supporting an intervention and make recommendations about their use, such as Grading of Recommendations Assessment, Development, and Evaluation (GRADE) or that used by the United States Preventative Services Task Force [3, 4]. These guidelines often consider different domains of research outcomes and quality, like the health outcomes, the research design, and potential for bias in the outcomes because of the research design. Based on these guides, health institutions, like the Preventative Services Task Force, make recommendations about the best interventions across a wide set of health conditions to assist providers and organizations in making clinical and policy-level decisions. To our knowledge, no equivalent set of criteria for implementation strategies are available. As such, it is difficult to discern the quality of evidence supporting an implementation strategy and whether strategies should be recommended to practitioners to support the implementation of EBIs.

Existing criteria, like GRADE, may serve as a valuable starting point for building criteria applicable to the field of implementation research [5]. Effectiveness research and associated evaluation criteria, which heavily emphasizes internal validity, considers the highest quality evidence to be from research designs like double-blind randomized control trials. In implementation research, internal validity tends to be more balanced with external validity so that the results are generalizable to target communities. With external validity in mind, implementation research is typically conducted in practice settings and involves assessment of the organizations and providers who will be impacted by the implementation strategy and subsequently the intervention under consideration. As a result, it is often inappropriate, impractical, and/or undesirable to leverage research designs like randomized controlled trials, because it is not possible to blind practitioners to the strategy and/or intervention or randomize at the unit of analysis [6–8]. These realities make direct application of intervention-level criteria inappropriate—necessitating criteria specific to the field [3].

### HIV and implementation research in the US

We describe our efforts to develop a set of criteria and evaluation process for implementation strategies to address the HIV epidemic in the United States. Improvements in the US HIV epidemic have been modest over the last two decades, with disparities among communities disproportionally affected by HIV increasing [9]. In an attempt to address HIV incidence, the Centers for Disease Control and Prevention have curated a repository of EBIs to support HIV prevention since the early 2000s and supported dissemination and implementation of a subset of these [10]. Furthermore, major biomedical advancements, such as pre-exposure prophylaxis (PrEP), have proven to be very effective at preventing HIV. Yet many of these interventions have not been widely implemented with equity to yield their intended benefit. Only an estimated 30% of individuals who would benefit from PrEP receive it, with growing disparities by race, gender, income, citizenship status, and intersectional marginalization [11–14]. Uptake and adherence remain suboptimal along the HIV care continuum (i.e., prevention, testing, diagnosis, linkage-to-care, and treatment), indicating, in part, failed implementation and opportunities to develop evidence-informed implementation strategies [11]. In 2019, the Ending the HIV Epidemic (EHE) Initiative was launched as a coordinated effort among several federal agencies to address HIV-related implementation problems. In alignment with EHE, the National Institutes of Health supported a number of mechanisms and projects to conduct research on implementation strategies [15]. With the growing mass of HIV-related implementation research has come an equally growing knowledge base of implementation strategies targeting multiple aspects of the HIV care continuum, in a wide scope of settings, evaluating various implementation outcomes [16].

In an effort to create, synthesize, and disseminate generalizable knowledge, the Implementation Science Coordination Initiative (ISCI) was funded by the National Institutes of Health to provide technical assistance in implementation research funded by the EHE Initiative, coordinate research efforts, synthesize literature through systematic reviews, develop tools to assist researchers, and disseminate research findings to researchers, policymakers, providers, and more [17, 18]. As part of this effort, we developed a tool to evaluate the quality of evidence of HIV-related implementation strategies to identify best-practice strategies that can promote effective implementation and uptake of EBIs. The long-term goal of this particular project is to accumulate, warehouse, and disseminate a collection of effective strategies that can be used by HIV practitioners nationwide to support the EHE Initiative.

## Methods

### Overview

We conducted the project in three phases: 1) a literature review in tandem with key informant interviews to generate initial criteria for our tool, 2) a modified Delphi to evaluate and revise our initial tool and criteria; 3) a pilot application of our rubric to a set of implementation research studies. Delphi data were collected from March 2022 to June 2023. Piloting occurred in the fall of 2023. Our data collection protocol was reviewed by the Institutional Review Board at Northwestern University and determined to be non-human subjects research. All data collection instruments have been included as a supplemental file (Supplemental File A), and data are available in a de-identified format from the first author on reasonable request. Methods and results are reported according to STROBE reporting guidelines (Supplemental File B).

### Key informant interviews and literature review

We first conducted a review of the scientific and grey literature of existing compilations of criteria for assessing EBIs. Google scholar was used to search for tools or criteria published in academic journals. To identify tools within the grey literature, we focused on federal institutions that frequently provide evidence-recommendations such as the US Preventative Task Force, the Centers for Disease Control and Prevention, and Health Services and Resources Administration. We utilized this literature to identify commonalities across tools, to review current debate on the philosophy of science as it relates specifically to implementation science, and to construct an interview guide for key informant experts with questions to elicit information about key differences between implementation research and existing tools. We also used the literature to identify experts who we then recruited for key informant interviews and our Delphi.

We recruited and interviewed a range of experts, including implementation scientists, HIV providers and implementers, representatives from related fields of public health research (e.g., quality improvement), and public health agency officials. All interviews were scheduled in the Spring of 2022, were approximately 30–45 min long, and were conducted by either VM or az. Briefly, the three main questions were: 1. Do you think existing criteria apply to implementation research studies? 2. What are essential indications of generalizability in implementation research? 3. What are ways to evaluate strategies with multiple components? Each question included follow up probes. Interviews were recorded and transcribed via Zoom. Participants were not given an incentive for participation. Two Ph.D.-level researchers with expertise in qualitative and mixed methods research performed an inductive, thematic process of analysis to explore patterns and categorize responses. Based on their responses, we iteratively developed a preliminary tool and criteria.

### Modified Delphi

#### Identification and recruitment of Delphi participants

We conducted an asynchronous, modified eDelphi with participants of similar expertise as our key informants in two rounds. Participants were recruited using snowball recommendations from those that were interviewed as key informants. Our eligibility criteria included fluent English speakers and those working in either HIV services research or those working in implementation research but in another field that may intersect with HIV, for example, mental health, substance misuse, social services, primary care, or women’s health. If participants were unable to complete the survey, an alternative contact could be recommended. After this first invitation, we sent semiweekly reminder emails for six weeks. A $10 gift card was given to participants for completing the first survey, and a $50 gift card was given to participants for completing the second survey.

#### Data collection and measures

The surveys were implemented using Qualtrics. The surveys were piloted with members of the ISCI research team to ensure question clarity. Each survey took participants approximately 45–75 min to complete.

#### First-round Delphi instrument

This survey consisted of 71 items. Participants were first introduced to the purpose of the project at large, which was to create a tool and set of criteria on which to evaluate HIV-related implementation science, and then to the specific goals of the Delphi, which was to generate consensus about which aspects of the tool were most important and least important and whether we had included all the elements that participants felt were necessary. The first portion of the survey gathered demographic and basic information about the participant (e.g., age, race, ethnicity, gender), characteristics of the participant’s work (e.g., I work primarily in… select all areas that apply”), as well as the participant’s experience in implementation research (e.g., How would you describe your knowledge level of implementation science?).

The second portion of the survey evaluated proposed domains for the tool (Overall Evidence of Effectiveness, Study Design Quality, Implementation Outcomes, Equity Impact, Strategy Specification, and Bundled Strategies) and corresponding criteria. Participants were asked to agree or disagree (Yes/No) with the adding/dropping/combining of domains; this was followed by an open-ended question asking why they agreed to said addition/dropping/combining (if applicable). This portion also contained two 5-point Likert-type scales asking participants to rank the domains in order from most important to least important. The third portion of the survey was aimed at gaining the participant’s opinion on the specific criteria (e.g., effect size and effect direction for implementation outcomes) within each domain. For each domain, the participant was asked if there were any criteria that needed to be added/dropped (Yes/No), followed by an open-ended question asking why they would like these items added/dropped (if applicable). The participant was then provided a 5-point Likert scale in which they ranked each item from “Very unimportant” to “Very important”. These questions were repeated for all criteria in all domains.

The final portion of the survey introduced the Levels of Evidence (Best Practice Strategy, Promising Strategy, Emerging Strategy, Undetermined Strategies, and Not Recommended Strategy) and their definitions. The participant was asked if there should be any adding/dropping/combining of the evidence levels (Yes/No), followed by an open-ended question asking why they would like these evidence levels to be added/dropped/combined (if applicable).

#### Second-round Delphi instrument

This survey consisted of 52 items. All participants from Round 1 were recruited for Round 2. Again, participants were reminded of the overall purpose of the project and the specific goal of the Delphi, which was to confirm changes to the tool made in response to the results of Round 1 and receive feedback. The first portion of the survey gathered the same demographic and basic information as in the first round. The second portion consisted of an overview of the updated tool, including definitions of the domains, criteria, and levels of evidence, and asked for feedback on changes made from the Round 1 results. For example, in the first round of the Delphi survey, participants responded that they would like for greater specificity within the criteria of the Study Design domain. As a response, we split this domain into two domains for Round 2: “Study Design” and “Study Rigor and Limitations.” We presented this change to the participant and asked them to agree or disagree with this change (Yes/No); if “No” was selected, this prompted an open-response question asking for further explanation. Lastly, we asked respondents to apply the criteria and give an evidence-level rating to a set of fictional cases of implementation research studies, allowing respondents to comment on the application and rating process.

#### Data analysis and management

Quantitative data were managed and analyzed in Excel. Quantitative data were analyzed descriptively, primarily as percent agreement or disagreement for domains, evidence levels, and individual criteria within domains. Qualitative data were analyzed in Dedoose software and Excel, using a rapid direct qualitative content analysis approach [19]. Qualitative data were analyzed by a Ph.D.-level researcher with qualitative research expertise and were intended to confirm or complement quantitative analyses.

### Pilot and application to PrEP implementation strategies

To ensure a high-quality process for reviewing literature and consistent application of criteria across the different evidence levels, we piloted and refined the tool with a set of implementation strategies designed to promote the uptake of evidence-based HIV services with members of ISCI, which consists of a mix of faculty, staff, and students holding degrees at the Bachelors, Masters, and PhD levels. VRM led two, hour-long trainings with, four Ph.D.-level members of the ISCI team who were also engaged in systematic reviews of HIV literature on how to apply the criteria. ISCI team members then applied the criteria to an existing set of eight papers reporting on implementation strategies designed to promote PrEP uptake coding a rating for each criteria and domain.  Studies were selected by VRM to represent the full range of evidence ratings and different points of the HIV care continuum (i.e., PrEP delivery, HIV testing, and retention in care for HIV treatment). We calculated agreement as a simple percentage of identical ratings between two coders out of the total number of criteria, domain ratings, and overall rating (40 items). In places where there was high disagreement, the tool was revised and refined to provide better guidance and instruction on how to apply specific criteria. In a final application of the tool, two coders, a Master’s and Ph.D. level member of the ISCI team, applied the criteria to an additional set of 18 implementation strategies designed to improve PrEP uptake and use identified through an existing systematic review [20] after a single hour-long training.

## Results

We report the primary results from each stage of our process as well as significant changes to the tool made at each stage.

### Literature review and key informant interviews

Our initial literature review yielded several existing rubrics, tools, criteria and processes for evaluating evidence supporting a specific intervention identified primarily in the grey literature developed by large institutions responsible for disseminating evidence-based interventions [5, 21]. Many had a similar structure of grouping criteria by domain (e.g., aspects of the research design or strength of the outcomes) and having different evidence ratings or levels (e.g., low, medium, high evidence strength). For example, the Centers for Disease Control and Prevention National Center for Injury Prevention Guide to the Continuum of Evidence of Effectiveness outlines six domains (i.e., effect, internal validity, type of evidence/research design, independent replication, implementation guidance, external and ecological validity) and seven evidence levels (harmful, unsupported, undetermined, emerging, promising direction, supported, and well supported) while the US Preventative Task Force has six domains or “factors” considered when generating an evidence level and five evidence levels or “grades” [21–23]. Our literature review also yielded several articles relevant to the generalization of evidence generated from implementation trials. For example, key considerations on whether effects are likely to be transferable from one context to another, balancing internal and external validity, and the need to consider equity in impact [21, 24–26].

We conducted a total of 10 interviews representing a mix of expertise in HIV services research, implementation research, and quality improvement research. Informants reflected on different potential domains (e.g., elements of the research design) and listed specific ways that they felt research and evidence quality differed in implementation research from clinical trials. Among factors highlighted were a need to consider the context and specification of strategies, criteria specific to implementation outcomes, and consideration of the equity impact of implementation strategies on the health outcome under consideration. Again, existing implementation science literature helped support and define domains like Proctor's recommendations for strategy specification to ensure that strategies are appropriately described as well as Proctor's implementation outcomes to define and describe implementation outcomes [1, 48].

Based on these collective results, conceptually, we modeled our initial tool by grouping criteria according to domain and having a series of evidence levels similar to many tools and criteria that we reviewed. We also worked to integrate current thinking and perspectives on implementation science, evidence, and generalizability into our tool from both the literature and key informant interviews. Briefly, we structured our initial tool along six domains: overall effectiveness, study design quality, implementation outcomes, equity impact, strategy specification, and a bundled strategies domain. Each domain included a set of criteria. For example, criteria for the implementation outcomes domain included operationalization of implementation outcomes; validity and reliability of measure used; significance and direction of effect for quantitative outcomes; and reported effects as beneficial, neutral, or harmful. We also developed and defined five evidence levels with associated recommendations: best practice strategy, promising strategy, emerging strategy, undetermined strategy, non-recommended strategy. As an example, promising strategies were described as demonstrating mostly positive outcomes that may need more rigorous examination to ensure they are having the intended effect or are generalizable to a wider context. Practitioners would be recommended to take caution when using a promising strategy in practice and ensure it is having a similar outcome as demonstrated in the original research.

### Modified Delphi

For the Delphi Round 1, we recruited from a pool of 68 experts. Two individuals responded stating their inability to participate, with one participant suggesting a replacement. Forty-one participants completed the survey, and two participants partially completed the survey for a total of 43 participants (63% response rate). For the Delphi Round 2, we recruited among the responders from Round 1 with no refusals to participate and no partial responses. Thirty participants in total completed the Round 2 survey (70% response rate). Respondent characteristics are provided in Table 1 for both Delphi Rounds. Briefly, one half of Respondents in both rounds self-identified as women (55.8%; 50% in rounds 1 and 2 respectively), with the majority white (83.7%; 80%) and not Hispanic or Latino (86%; 100%). Most respondents worked in academic settings (81.4%; 80%), with most working in HIV in round 1 but not round 2 (83.7%; 36.7% respectively). The highest number respondents had 11–20 years of experience in their area of expertise (44.2%; 43.3% respectively), and three quarters reported experience with leading implementation research projects (76.7%; 73.3%). Both complete and partially complete responses were included in subsequent analyses.

**Table 1:** Participant characteristics

	**Round 1**		**Round 2**	
	N	(%)	N	(%)
I work primarily in… (Select all that apply)				
Clinical settings	15	(34.9)	2	(6.7)
Public health settings	12	(27.9)	3	(10.0)
Academic settings	35	(81.4)	24	(80.0)
Federal government	5	(11.6)	3	(10.0)
Other	1	(2.3)	2	(6.7)
I spend a significant percent of my time (select all that apply)				
Providing services directly to patients or clients	10	(23.3)	6	(20.0)
Researching interventions, practices, or policies for scientific purposes	39	(90.7)	24	(80.0)
Evaluating interventions, practices, or policies for organizational quality improvement	23	(53.3)	10	(33.3)
Determining and providing funding for health services	7	(16.3)	2	(6.7)
Developing or implementing policies that influence the provision of health services	6	(14.0)	4	(13.3)
Other	1	(2.3)	0	(0.0)
I work in the following areas… (Select all that apply)				
HIV	36	(83.7)	11	(36.7)
Mental Health	18	(41.9)	10	(33.3)
Primary Care	22	(51.2)	10	(33.3)
Substance Misuse	10	(23.3)	6	(20.0)
Women's Health	14	(32.6)	8	(26.7)
Social Services	7	(16.3)	1	(3.3)
Another Area of Health	11	(25.6)	9	(30.0)
How would you describe your level of knowledge of implementation science?				
I know almost nothing or only the basic components of implementation science	2	(4.7)	1	(3.3)
I know a moderate amount about implementation science	12	(27.9)	5	(16.7)
I know a lot about implementation science	29	(67.4)	24	(80.0)
How many years of experience do you have in your field?				
0–10 years	5	(11.6)	4	(13.3)
11–20 years	19	(44.2)	13	(43.3)
21–30 years	12	(27.9)	5	(16.7)
31 years of more	7	(16.3)	8	(26.7)
What is your race? (Select all that apply)				
Asian	5	(11.6)	6	(20.0)
Black or African American	2	(4.7)	0	(0.0)
White	36	(83.7)	24	(80.0)
American Indian, Alaska Native, Native Hawaiian, Pacific Islander, or Other	2	(4.7)	3	(10.0)
Prefer not to respond	1	(2.3)	1	(3.3)
What is your ethnicity?				
Hispanic or Latino	1	(2.3)	0	(0.0)
Not Hispanic or Latino	37	(86.0)	30	(100)
No response	5	(11.6)	0	(0.0)
How do you describe yourself? (Select all that apply)				
A man	17	(39.5)	13	(43.3)
A woman	24	(55.8)	15	(50.0)
Nonbinary	1	(2.3)	1	(3.3)
Prefer not to respond	1	(2.3)	1	(3.3)
Total	43	(100)	30	(100)

#### Delphi round 1

Table 2 presents the quantitative outcomes regarding whether the participant believed that domains should be added, dropped, or combined. More than half (58%) of participants thought no new domains should be added, while 44% of participants thought domains should be dropped or combined. When examining the evidence levels, 79% of individuals felt that no additional evidence levels were needed, while 47% thought one or more of the evidence levels could be dropped or combined.

**Table 2:** Confirming domain and evidence level changes

Delphi Round 1 Results						
Should domains be…	Yes		No		Missing	
	N	(%)	N	(%)	N	(%)
Added?	17	(39)	25	(58)	1	(2)
Dropped?	6	(14)	35	(81)	2	(5)
Combined?	13	(30)	29	(67)	1	(2)
Should evidence levels be…	Yes		No		Missing	
	N	(%)	N	(%)	N	(%)
Added?	5	(12)	34	(79)	4	(9)
Dropped?	8	(19)	31	(72)	4	(9)
Combined?	12	(28)	29	(67)	2	(5)
**Delphi Round 2 Results**						
Do you agree with the revisions?	Yes		No		Missing	
	N	(%)	N	(%)	N	(%)
Study Design and Limitations	25	(85)	5	(15)	0	(0)
Dropped Bundled Strategies	26	(87)	4	(13)	0	(0)
Equity Impact	23	(75)	7	(25)	0	(0)
Combined Evidence Levels	27	(90)	3	(10)	0	(0)

Table 3 summarizes open-ended responses with example quotes for domains and evidence levels that were commented on most often. When reviewing the qualitative responses of those who indicated a domain should be added, most respondents suggested adding specific criteria or wanted greater clarity in how the domains and criteria within domains were defined. For example, regarding the equity domain, individuals desired greater clarity, operationalization, and description of how equity is being considered and evaluated. Of these, four sought greater clarity of equity-related outcomes, and six recommended inclusion of equity metrics or different ways of operationalizing equity. Three participants felt equity should be examined in combination with implementation outcomes. Three suggested greater consideration of community partnership development and inclusion of the target population in the development of the strategy or design of a study. Finally, participants recommended combining promising, emerging, and/or undetermined as levels of evidence and better specifying and operationalizing the levels.

**Table 3:** Delphi open-ended responses

Round 1 Suggestions	
Theme	Quote
Combine Equity with other Domains	"Not to drop equity—it should be somewhere -, but equity and reach (from implementation outcomes have considerable overlap). This could be a single implementation outcome domain."
Combine Bundled Strategies with Other Domains	“Nearly all implementation interventions are complex health interventions and hence bundled or multi-component. Any considerations related to bundled strategies should be generalized and applied to all strategies ….”
Clarify Overall Evidence of Effectiveness	"Overall evidence of effectiveness is generally understood as average effect size estimates, which are largely meaningless and unhelpful when studying complex health interventions.… strong, high-quality designs for complex health interventions require application of the core function/form framework, allowances for adaptation and tailoring and assessment of fidelity to function (not form).. Research answering the simple (simplistic) question "is intervention X effective" offers little value in implementation science, insofar as the dominant answer is 'sometimes' or 'it depends'."
Merge Medium Levels (promising/ emerging)	“I struggle a little with the distinction between Promising Strategies and Emerging Strategies. I suspect the distinction might be between the number of studies? but the conclusion seems to be the same regardless.”
Round 2 Open-ended Responses on Equity Domain Changes	
Hard no on equity	"I still don't see why this isn't considered as an implementation outcome. It seems like a normative decision to separate the domain…This just seems like an unnecessary complexity."
Soft no on equity with suggested changes	"I agree with keeping it separate but think there are two separate domains: (1) the actual reach to specific populations. This can be covered elsewhere and (2) the intentionality of the strategy to reach a specific marginalized population (including involving marginalized individuals/population representatives in strategy development and research design). I think this is very reasonable to have as a separate domain. I would give examples of target populations, but not define them or leave it to the CDC to define. Some of the most marginalized populations may be identified through community engaged research and implementation science Might consider using a more well-established definition of health equity research–for example, something from CDC or NIH. Also seems like the partner engagement would be more under engagement (broadly speaker) not explicitly called out under health equity…."

Briefly, we revised the structure of our tool along five domains: study design, implementation outcomes, study rigor and limitations, strategy specification, and equity impact. These domains each included a revised set of criteria. For example, based on the recommended additions to the study design and rigor domain, we split this domain into two domains: 1) study design; and 2) study rigor and limitations. We considered several of the comments on dropping equity but ultimately opted to keep this domain, relax the criteria, and heavily refine the description. Other cross-cutting changes included combining the criteria for bundled strategies and strategy specification. We combined two of the evidence levels (emerging and undetermined) and revised the definitions to include: best practice, promising practice, needs more evidence, and harmful.

#### Delphi round 2

For the second round of the Delphi, we asked respondents to confirm major changes to the tool based on the first round of the Delphi (Table 2), and have respondents evaluate our proposed process for applying the criteria. Most respondents agreed with changes to the domains and evidence levels although there remained some commentary on the equity domain. When examining the open-ended responses among those disagreeing with the changes to the equity domain, we grouped responses into individuals that did not agree with the domain (i.e., a hard no to the revisions) and others who still had additional suggestions for the domain but approved of the domain overall (i.e., a soft no with suggested revisions; Table 3). Based on these responses, we finalized the domains and made several additional adjustments to the definitions of equity including defining which target populations can be considered in determining whether the strategy has a positive equity impact or not. Finally, we revised our process for applying the rubric based on the recommendation to apply the criteria across each domain in addition to giving an overall rating. While this did increase time in the review process, this change allowed us to still provide information on how strategies rate across all domains, enabling researchers and practitioners to compare how strategies rate on different domains or select a strategy that is strong in a specific domain, like equity supporting for example.

### Pilot application to PrEP implementation strategies

To ensure a consistent, high-quality process for applying criteria to research studies examining implementation strategies, we initially piloted the rubric with eight existing studies on implementation strategies to promote the uptake of evidence-based HIV services including PrEP, HIV testing, and retention in care [27–34]. At the conclusion, we were able to achieve 90% reliable application of the criteria, resulting in dropping some criteria and clarifying other criteria and their application. Two members of the ISCI team then applied the rubric to a set of 18 implementation strategies identified through an ongoing systematic review designed to promote uptake of PrEP in a second pilot application, achieving 98% reliability and taking approximately 15–30 min per article.

Among the 18 strategy studies, summarized in Table 4, one was assigned an overall rating as Best Practice and the remaining were assigned as Needs More Evidence. The primary domains where strategies failed to exceed the Needs More Evidence criteria were in Research Design as well as Study Rigor and Limitations. This was largely because these studies only utilized post-implementation assessment, were intended as pilot or feasibility studies, or were conducted only at a single site. Given the early state of the implementation research related to PrEP implementation in the US, we felt that this mix of ratings was relatively appropriate. While the domains that have parallels in other rating systems resulted in relatively low ratings among our studies, we observed a good mix of ratings on domains unique to our tool and implementation research (i.e., strategy specification and equity) at the Best, Promising, and Needs More Evidence levels, suggesting these domains are sufficiently discerning among the existing set of studies.

**Table 4:** Rubric and criteria application to 18 implementation science studies focused on PrEP

First Author	Year	Article Title(s)	Study Design	Outcomes	Rigor	Specifica-tion	Equity	Overall
Brant	2020	Integrating HIV Pre-Exposure Prophylaxis into Family Planning Care: A RE-AIM Framework Evaluation [29]	Needs More	Best	Needs More	Best	Promising	Needs More
Buchbinder	2019	Getting to Zero San Francisco: A Collective Impact Approach [35]	Needs More	Needs More	Needs More	Needs More	Best	Needs More
Bunting	2019	A Guide for Designing Student-Led, Interprofessional Community Education Initiatives About HIV Risk and Pre-Exposure Prophylaxis [36]	Best	Needs More	Needs More	Best	Promising	Needs More
	2020	Using a student-led, community-specific training module to increase PrEP uptake amongst at-risk populations: results from an exploratory pilot implementation [37]						
Burns	2021	Meet Me Where I Am: An Evaluation of an HIV Patient Navigation Intervention to Increase Uptake of PrEP Among Black Men Who Have Sex with Men in the Deep South [27]	Needs More	Best	Needs More	Best	Needs More	Needs More
Chen	2014	Clinical effectiveness and cost-effectiveness of HIV pre-exposure prophylaxis in men who have sex with men: risk calculators for real-world decision-making [30]	Needs More	Needs More	Promising	Needs More	Promising	Needs More
Clement	2019	Advancing the HIV Pre-Exposure Prophylaxis Continuum: A Collaboration Between a Public Health Department and a Federally Qualified Health Center in the Southern United States [28]	Needs More	Best	Best	Best	Best	Needs More
Coleman	2020	Integrated Pharmacy and PrEP Navigation Services to Support PrEP Uptake: A Quality Improvement Project [38]	Best	Best	Best	Best	Best	Best
Gregg	2020	Implementation of HIV Preexposure Prophylaxis in a Homeless Primary Care Setting at the Veterans Affairs [39]	Needs More	Best	Needs More	Best	Best	Needs More
Havens	2019	Acceptability and feasibility of a pharmacist-led HIV pre-exposure prophylaxis (PrEP) program in the Midwestern United States [32]	Needs More	Best	Needs More	Promising	Promising	Needs More
Horack	2020	Pre-Exposure Prophylaxis in a Reproductive Health Setting: A Quality Improvement Project [33]	Needs More	Needs More	Needs More	Needs More	Needs More	Needs More
Hoth	2019	Iowa TelePrEP: A Public-Health-Partnered Telehealth Model for HIV Pre-Exposure Prophylaxis (PrEP) Delivery in a Rural State [40]	Needs More	Best	Needs More	Best	Promising	Needs More
Khosropour	2020	A Pharmacist-Led, Same-Day, HIV Pre-Exposure Prophylaxis Initiation Program to Increase PrEP Uptake and Decrease Time to PrEP Initiation [41]	Needs More	Best	Needs More	Promising	Promising	Needs More
Lopez	2020	Implementation of pre-exposure prophylaxis at a community pharmacy through a collaborative practice agreement with San Francisco Department of Public Health [42]	Needs More	Best	Needs More	Best	Best	Needs More
Pathela	2020	The HIV Pre-exposure Prophylaxis (PrEP) Cascade at NYC Sexula Health Clinics: Navigation is the Key to Uptake [43]	Needs More	Best	Needs More	Needs More	Promising	Needs More
Roth	2020	Integrating HIV Preexposure Prophylaxis With Community-Based Syringe Services for Women Who Inject Druges: Results from the Project SHE Demonstration Study [44]	Needs More	Best	Needs More	Best	Promising	Needs More
Saberi	2018	A Simple Pre-Exposure Prophylaxis (PrEP) Optimization Intervention for Health Care Providers Prescribing PrEP: Pilot Study [45]	Needs More	Best	Needs More	Best	Needs More	Needs More
Tung	2018	Implementation of a community pharmacy-based pre-exposure prophylaxis service: a novel model for pre-exposure prophylaxis care [46]	Needs More	Best	Needs More	Best	Needs More	Needs More
Wood	2018	Project ECHO: telementoring to educate and support prescribing of HIV pre-exposure prophylaxis by community medical providers [47]	Best	Needs More	Needs More	Promising	Needs More	Needs More

A summary of major changes to the rubric and criteria are summarized in Table 5. The final domains and evidence-levels are provided in Table 6, and a summary of the criteria by domain at each evidence level is provided in Table 7. The final tool with domains, criteria, evidence levels, and application instructions are available as a supplement (Supplemental file C).

**Table 5:** Summary of rubric and criteria revisions after each development phase

Initial review and Interviews	Delphi Round 1	Delphi Round 2	Piloting
• Initial proposed domains: overall effectiveness, study design quality, implementation outcomes, equity impact, strategy specification, & bundled strategies • Initial proposed evidence levels: best practice, promising, emerging, undetermined, & non-recommended strategy • Initial proposed criteria for each domain	• Dropped overall effectiveness domain • Split study design quality domain into study design and rigor and limitations domain • Revised equity domain description • Combined emerging and undetermined evidence levels and renamed as more evidence needed • Reduced criteria across all domains at all evidence-levels	• Confirmed domains • Revised equity domain descriptions • Confirmed evidence levels • Reduced criteria across all domains at all evidence-levels • Revised application process	• Revised application process • Revised criteria definitions

**Table 6:** Descriptions of criteria domains and evidence levels

Criteria domains	
Domains	Descriptions
Study Design	The elements of study design(s) used to evaluate a strategy, primarily the use of a comparison group and assessment before (pre) and after (post) a strategy is used
Implementation Outcomes	The effect and effect direction of deliberate and purposive actions to implement new treatments, practices, and services
Rigor & Limitations	Aspects of the study design that may limit the validity or generalizability of the results
Strategy Specification	The level of specificity about the strategy for purposes of reproducibility
Equity Impact	The impact of the strategy among target populations disproportionately impacted by the HIV epidemic
**Evidence Levels**	
Evidence Levels	Descriptions
Non-Recommended	Strategies demonstrate a harmful effect Strategies would not be recommended for practice or scientific investigation
Needs More Evidence	- Strategies demonstrate null results or positive results from preliminary research Strategies would be recommended for additional research with more rigorous research designs
Promising	Strategies demonstrate positive outcomes but may be limited on some aspects of criteria domains Strategies would be recommended to practitioners but may require careful monitoring to ensure similar outcomes
Best	Strategies demonstrate positive outcomes from rigorous research Strategies would be recommended to practitioners for the barriers they are intended to address

**Table 7:** Summary of domain criteria for each rating level

Best Practice Strategies	Promising Strategies	More Evidence Needed	Non-Recommended Strategies
○ At least one study with a pre/post assessment *or* a comparison group ○ Positive primary implementation outcome effect ○ No major limitations to study rigor ○ Strategy well specified ○ Equity impact demonstrated	○ At least one study with a pre/post assessment *or* a comparison group ○ Positive primary implementation outcome effect ○ Some limitations to study rigor ○ Strategy mostly well specified ○ Equity impact considered ○ May not improve but does not exacerbate inequities	○ Pilot or feasibility, post-assessment only with no comparison group, or simulation study ○ Null primary implementation outcome effect ○ Some limitations to study rigor ○ Strategy is not well specified ○ Equity impact not demonstrated or considered	○ Harmful implementation outcome effects reported ○ Worsening inequity impact reported

## Discussion

To our knowledge, this is the first set of criteria to evaluate evidence for implementation strategies and serve as a basis for recommendations to practitioners. Our Best Practice tool was initially informed by existing criteria and interviews, refined by a Delphi, and then piloted with implementation strategies. This process yielded a rating scale (i.e., best, promising, needs more evidence, and harmful) and domains (e.g., study design, implementation outcomes, rigor and limitations), which are common to other tools and rubrics. Yet, implementation research’s system-level focus required tailoring to our rubric for some domains, like study design and outcomes, and the development of entirely new domains, specifically strategy specification and equity. To help define the criteria for the domains, we used results from key informant interviews and existing implementation science literature to help ensure appropriateness for the field [1, 6, 48]. As a specific example of tailoring, we have outlined criteria for the research design domain that considers the realities of where implementation research is conducted and does not require blinding or randomization for strategies to be considered the highest rating. While these helped provide structure and specific criteria at each of the evidence levels, in conducting the pilot we noted missing information which sometimes made it difficult to evaluate the research. We recommend using Standards for Reporting Implementation Studies (StaRI) guidelines as well as Proctor’s recommendations for strategy specification when reporting implementation research to help ensure the needed details to evaluate the research are reported and available for potential practitioners to understand what resources and efforts are needed for implementation strategies [1, 49].

In addition to being a new resource for implementation science, to our knowledge this is also the first evidence rating criteria that considers the potential to improve equity in a health issue. Because implementation science directly impacts communities with the potential to improve or exacerbate inequities, HIV included, experts reiterated that equity was a critical domain to include. However, our work among participants, who primarily identified as white and non-Latin, demonstrates a lack of consensus in the implementation science field about what equity in implementation science means. We also encourage continued discussion within the implementation science community that includes diverse perspectives to help foster consensus and bring additional attention to this problem.

For the Best Practices Tool, the criteria within the Equity domain emphasizes community engagement in the research process, a research focus on populations experiencing inequities, as well as equity in outcomes for the Best Practice evidence level rating. as a means to These criteria encourage attention to and improvement in HIV-related inequities as many in the field have advocated [50–52] with additional, more relaxed criteria for lower evidence ratings. However, we recognize that no single implementation strategy (or intervention) is going to adequately address the deeply rooted structural determinants, like racism and homophobia, which keep inequities entrenched. Implementers who are interested in utilizing strategies may wish to consider additional factors that are relevant to their specific contexts, like whether communities they serve are reflected in the strategies they are considering or whether the strategy responds to the determinants driving inequity in their context. However, it is our hope that by including equity improvement as criteria to be considered the highest quality research, we can bring additional attention to and encourage equity in HIV outcomes in the US.

Our tool and criteria are designed to discern among studies for which there is best evidence specific to HIV implementation strategies in the US, which is a rapidly growing field, rather than having an absolute threshold of effectiveness that studies must meet. There are other heath areas, such as cancer and global HIV implementation research, for which there are more studies leveraging more rigorous research designs to evaluate implementation strategies [53, 54]. If applied in these areas, it may be more appropriate to have more stringent criteria to adequately discern among studies for which there is relatively good evidence compared to those which would need additional study. We encourage others who may consider using this tool in their area of implementation science to consider adapting the specific criteria within each of the domains and at each of the evidence-levels to ensure that it appropriately discerns among available studies before routine application. Continuing with the example of more rigorous research designs, it may be appropriate to require better replication of results or more diverse settings than we have incorporated into our specific criteria. However, we would suggest that the overall structure of the tool, specifically the domains and recommendation levels could remain the same regardless of the health field. Conversely, we received many suggestions for more stringent criteria that participants felt like should be included that we were not able to include because it would have resulted in few-to-no strategies identified as best practice. US focused HIV implementation science is still in its adolescence, with many pilots and full-fledged trials underway but not yet published. It is our hope that in the future, we will be able to include more stringent criteria within the rubric so that the needed evidence quality improves over time within HIV implementation research.

There are some notable limitations to the processes used to develop the Best Practice Tool and the criteria themselves. We used a modified eDelphi approach to develop the rubric and criteria with some loss to follow up from the first to the second round of the Delphi, particularly among HIV service providers which may mean the results are not sufficiently representative of this context. However, we did retain many individuals working in settings where HIV services intersect, like substance misuse, mental health, and social services. Our use of a modified Delphi method did not result in consensus, but instead resulted in an approximation of consensus. In addition, we were not able to elicit the opinions about the appropriateness of the tool from the perspective of front-line HIV service implementers on balance with those of the research community. We hope to address this in future iterations of this work.

We envision several future directions for this tool with implications for both researchers and practitioners that will advance the goals of ISCI and support the EHE Initiative. Systematic reviews of HIV-related implementation strategies are currently underway through ISCI [55]. The next phase will entail applying these criteria to implementation strategies identified through these reviews and developing a compendium of strategies. We recognize that a rating and recommendation is not sufficient to support uptake, and we also have a complementary dissemination effort underway to provide the needed information and materials for wide adoption and scale up which will be available on the ISCI website [18]. Our criteria and rating system will also yield benefits for researchers conducting HIV implementation research. Through our efforts, we will also identify strategies that hold promise but would benefit from additional research and additional evidence supporting their effectiveness. Researchers can also use these criteria in designing studies of new strategies so that they can score better on these criteria.

## Conclusion

For practitioners to fully benefit from research developing and testing implementation strategies targeting HIV services, clear evaluation criteria and recommendations are needed to assess which strategies are the most likely to have benefit and impact. We developed domains and criteria appropriate to evaluate evidence quality in HIV-related implementation strategies. This rubric includes recommendations for practitioners about strategies for which there is best evidence and recommendations for research about strategies for which more evidence is needed. Establishing criteria to evaluate implementation strategies advances implementation science by filling a much-needed gap in HIV implementation research which can be extended to other areas of implementation science.

### Supplementary Information

Supplementary Material 1.

Supplementary Material 2.

Supplementary Material 3.

## Data Availability

The Delphi dataset generated during the current study available from the corresponding author on reasonable request.
