# Supplementary material for: Establishing evidence criteria for implementation strategies in the US: a Delphi study for HIV services
Source: Implement Sci. 2024 Jul 15;19:50. doi: 10.1186/s13012-024-01379-3 (PMC11251241; doi:10.1186/s13012-024-01379-3)
Supplement: Supplementary file 1 — Supplementary Material 1. [file 13012_2024_1379_MOESM1_ESM.docx]

Supplement A – Study Instruments

ISCI Determining Best Practices Key Informant Interviews

Interview Guide

Developed: February 2022

Hi, my name is _______. It’s great to meet and speak with you today. I am a _______ with the Implementation Science Collaboration, Coordination, and Consultation Initiative (or ISCI), which is an NIH-funded center at Northwestern University. We are working to promote the uptake or implementation of beneficial HIV services in practice. Even though you may or may not have experience in (select one or both according to interviewee: HIV or Implementation Science), we think your perspective and insight would be valuable. We will take notes, but we will be recording our conversation in case I miss anything important or would like to revisit our conversation. This recording and the interview transcript will only be used to inform our later Delphi process. The interview should take about 30 minutes, and we’re grateful for you taking the time out of your day to speak with us. Do you have any questions before we begin?

- For KIs who are not implementation scientists:
  - As someone involved in HIV research or service prevision you know that evidence-based interventions and practices such as PrEP or ART are often poorly implemented, if at all. Implementation research seeks to understand and address barriers that slow or halt implementation of evidence-based interventions and practices. Our focus in this interview is on implementation strategies or the things researchers, providers, and others do to help introduce and promote intervention implementation. Examples can include such as restructuring organizations or clinical care, providing new training to staff, partnering with other agencies, creating incentives for staff or clients, and so forth. Implementation research is designed to test them scientifically to determine whether they work or not in addressing barriers and supporting implementation.

As part of this initiative, we are working to establish criteria to evaluate implementation strategies and to determine which strategies to recommend to researchers and providers alike for evidence-based HIV prevention and treatment services. Within the context of HIV prevention and treatment with the goal of Ending the HIV Epidemic by 2030 the field has worked rapidly to develop and test implementation strategies. At this point in time, we have a wealth of evidence on implementation strategies with multiple studies trialing the same or similar implementation strategies. For example, we have numerous trials of strategies to support the implementation of pre-exposure prophylaxis (or PrEP, a daily pill to prevent HIV for those who are at risk for acquiring HIV) like conducting ongoing training, altering patient or consumer fees, or intervening with patients or consumers to enhance uptake. However, we don’t yet have any clear criteria for identifying or recommending these implementation strategies to practitioners.

1. [10 minutes] You likely know concepts, criteria and tools commonly used for evaluating scientific evidence and determining whether it should be recommended. For instance, GRADE, evaluates evidence on whether there is an effect, what’s the size of the effect, internal validity, and potential for bias. Do you think these kinds criteria can be applied to evaluations of implementation strategies?
   1. Probes: If yes, why?
   2. [If not mentioned]: What about
      - Importance or significance of the health outcome
      - #’s of studies
      - and their design
      - Risk of bias
      - Use of control groups
      - Effect, and effect size.
      - Evidence from practice/colleagues
      - Evidence from studies in other diseases
   3. Would any modifications be needed for applying them to implementation strategies?
   4. If no, why not? Are there other criteria you would suggest? Are there specific tools you like?
   5. How much evidence would you like to see? For example, to consider a strategy evidence-based, would you need to see a multisite RCT and a certain number of controlled clinical trials? Or other study designs and methods?
2. [10 minutes] Researchers and practitioners have noted that often, practitioners’ and providers’ main concerns are whether an innovation and implementation strategy will work within their particular setting. The rest of the questions are focused on what helps us know whether evidence applies across different situations and contexts, like generalizability or external validity. In your opinion, what contextual factors are important indications of generalizability and are critical to take into account for evaluating and recommending implementation strategies?
   1. [If not mentioned]: What about
      - Specific settings?
      - Who is actually responsible for enacting or delivering the strategy?
      - The intended or actual outcome of the strategy?
      - Cost of the strategy?
      - Feasibility of the strategy?
      - The intended people or targets of the strategy?
      - The implementation barriers addressed?
   2. Which do you think are the most important, if any:
   3. How much evidence would you like to see?
3. [5 minutes] As you may know, many strategies have multiple components (functioning as a bundle of strategies), which can address different implementation barriers and have different intended outcomes. How would you recommend we evaluate such strategies that aren’t just a single action by a single group of actors?
   1. What if we can’t separate the effects?
   2. What if there’s only one instance examining the particular make-up of a bundle?
4. We’ve reached the end of our questions, but is there anything I haven’t asked today that you want to share, or feel is important for us to take into consideration?

The next step in our process is to do a Delphi project based on your responses which will help generate consensus about the topics we talked about today. We would love for you to participate in that if you have time, but we are also interested in your recommendation on who else might be good to invite. Do you have anyone that you would recommend? What is the best way to contact to them?

Again, thank you so much for speaking with me today. Your thoughts will help us greatly in moving forward with figuring out best practices or a guide in evaluating implementation strategies for HIV research.

Delphi Survey Round 1

Introduction

      The Implementation Science Coordination Initiative (ISCI) team at Northwestern University works to promote high-quality implementation science and create opportunities to synthesize generalizable knowledge in HIV research and practice funded through the Ending the Epidemic (EHE) Initiative. For those not familiar with implementation science or its application in practice-based settings, implementation science is the systematic study of how to promote adoption and integration of evidence-based practices, interventions, and policies into routine health care and public health settings, to improve the impact on population health.

Our focus in this survey is on implementation strategies or the things researchers, providers, and others do to help introduce and promote intervention implementation. Examples can include restructuring organizations or clinical care, providing new training to staff, partnering with other agencies, creating incentives for staff or clients, and so forth. Implementation research tests these strategies scientifically to determine whether they work or not in addressing barriers and supporting implementation. However, we do not have criteria for evaluating quality of science supporting implementation strategies or for making recommendations to practitioners about which strategies are most beneficial.  To begin the work of establishing criteria for strategies related to HIV interventions, we conducted a review of the scientific literature. We then interviewed a range of key informants, including implementation scientists, HIV providers and implementers, and representatives from related fields. Based on their responses, we have started to develop a preliminary rubric for evaluating the quality of implementation strategy research. The following survey will ask you questions about different aspects of our rubric to help generate consensus about which aspects of the rubric are most important, least important, and whether we have included all the elements that you feel are important. 

First, we would like to know a bit more about your experience with implementation research and your area of expertise (* indicates a required response).

1. I work primarily in (select all that apply)*

- Clinical settings
- Public health settings
- Academic settings
- Other

2. Please specify

________________________________________________________________

3. I spend a significant percent of my time (select all that apply)*

- Providing services directly to patients or clients
- Researching interventions, practices, or policies for scientific purposes
- Evaluating interventions, practices, or policies for organizational quality improvement
- Determining and providing funding for health services
- Developing or implementing policies that influence the provision of health services
- Other

4. Please specify

________________________________________________________________

5. I work in the following areas (select all that apply)*

- - HIV
  - Mental Health
  - Substance Misuse
  - Social Services
  - Primary Care
  - Women's Health
  - Another area of health

6. Please specify the other area of health

________________________________________________________________

7. How would you describe your level of knowledge of implementation science (e.g., theories, frameworks, implementation outcomes)?*

- I know almost nothing about implementation science
- I know about the basic components of implementation science
- I know a moderate amount about implementation science
- I know a lot about implementation science

8. What would you say is your level of experience in implementation research? (Select all that apply)*

- I have not formally been involved in implementation research
- I have supported implementation research projects
- I have led implementation research projects
- I have been a partner in implementation research

9. How many years of experience do you have in your field?

- 0-10 years
- 11-20 years
- 21-30 years
- 31 years or more

10. What is your race? (Select all that apply)

- American Indian or Alaska Native
- Asian
- Black or African American
- Native Hawaiian or other Pacific Islander
- White
- Other
- Prefer not to respond

11. What is your ethnicity?

- Hispanic or Latino
- Not Hispanic or Latino

12. How do you describe yourself? (Select all that apply)

- A man
- A woman
- Nonbinary
- Prefer not to respond

**Evaluation Domains**

To help evaluate the scientific evidence for individual implementation strategies we are proposing five broad domains: overall evidence, study design quality, implementation outcomes, strategy specification, equity impact, and special considerations for multi-component or bundled strategies. We have included a short description and specific items for each domain below. Please read through the table and answer the questions that follow:

| Evaluation Domains | Description | Specific items included |
| --- | --- | --- |
| Overall Evidence of Effectiveness | The overall evidence supporting a strategy, including the number of studies, the consistency of the effects on implementation outcomes, and generalizability of a strategy across contexts. | - The number of studies - The mix of study designs - Consistency of implementation outcome effects - Magnitude of effects - Variety of settings represented - Equity impacts - Level of strategy specification across studies |
| Study Design Quality | The quality of the study designs used to evaluate a strategy. | For Quantitative Studies   - Use of a control arm or comparison group - Pre/post implementation outcome measurement - Approaches to bias control (i.e., randomization, sampling) - Extent of limitations - Adequacy of power   For Qualitative Studies   - Rationale for participant selection - Rationale for data collection approach - Rationale for data analysis approach - Description adequacy for data collection, management, and transcription - Description adequacy for data analysis, codes, and code definitions. - Use of intercoder reliability techniques. - Extent of limitations   For Mixed Method Studies   - Extent to which methods complement or support one another |
| Implementation Outcomes | The type of implementation outcomes measured, operationalization of the implementation outcomes, and quality of measurement of the implementation outcomes. | - Operationalization of implementation outcomes - Validity and reliability of measures used - Quant only: Significance and direction of effect - Qual only: Reported effects, either beneficial, neutral, or harmful. |
| Equity Impact | The impact of a strategy on promoting health equity. | - Consideration of equity in research design - Effect on equity-related outcomes |
| Strategy Specification | The rationale for selecting a strategy (i.e., theory, identified barriers, and mechanisms). The quality of specification of a strategy for future practice or research or for replicability of a strategy. | - Theoretical justification for strategy selection - Rationale for how the strategy works and why it should be effective. - Level of detail regarding the context in which the strategy was tested - Level of detail regarding the barriers and facilitators addressed by the strategy. - Level of detail regarding the implementation strategy itself |
| Bundled strategies | Special considerations for bundled strategies (i.e., multiple strategies that are delivered together), such as justification for bundled strategies and ability to discern effects of individual components. | - Theoretical justification for strategy selection - Analyses demonstrating that strategies are more effective when delivered together - Analyses demonstrating the relative value of individual strategies tested |

13. Do you think that there should be other domains?

- No
- Yes

14. If yes, what would you add?

________________________________________________________________

15. Do you think any of these domains should be dropped?

- No
- Yes

16. If yes, which one(s)?

________________________________________________________________

17. Should any of these domains be combined?

- No
- Yes

18. If yes, which ones?

________________________________________________________________

19. In your opinion, how important or unimportant are each of these domains

|  | Very unimportant | Somewhat unimportant | Neither important or unimportant | Somewhat important | Very important |
| --- | --- | --- | --- | --- | --- |
| Overall Evidence of Effectiveness |  |  |  |  |  |
| Study Design Quality |  |  |  |  |  |
| Implementation Outcomes |  |  |  |  |  |
| Equity Impact |  |  |  |  |  |
| Strategy Specification |  |  |  |  |  |
| Bundled strategies |  |  |  |  |  |

20. Please rank the domains in order of importance from MOST important to LEAST important with **1 being MOST important.**

______ Overall Evidence of Effectiveness

______ Study Design Quality

______ Implementation Outcomes

______ Equity Impact

______ Strategy Specification

______ Bundled Strategies

21. Is there anything else about the domains for assessing implementation strategies that you would like to share?

________________________________________________________________

________________________________________________________________

22. We are also interested in your opinion about the specific items included in each domain.

23. We describe the domain **Overall Evidence of Effectiveness** as the overall evidence supporting a strategy, including the number of studies, the consistency of the effects on implementation outcomes, and generalizability of a strategy across contexts. We propose this would include the following specific items:

- The number of studies
- The mix of study designs
- Consistency of implementation outcome effects
- Magnitude of effects
- Variety of settings represented
- Equity impacts
- Level of strategy specification across studies

24. Are there any items you would add?

- No
- Yes

25. If yes, what?

________________________________________________________________

26. Are there any you would drop?

- No
- Yes

27. If yes, what?

________________________________________________________________

28. In your opinion, how important or unimportant are these items for the Overall Evidence of Effectiveness domain they represent

|  | Very unimportant | Somewhat unimportant | Neither important or unimportant | Somewhat important | Very important |
| --- | --- | --- | --- | --- | --- |
| The number of studies |  |  |  |  |  |
| The mix of study designs |  |  |  |  |  |
| Consistency of implementation outcome effects |  |  |  |  |  |
| Magnitude of effects |  |  |  |  |  |
| Variety of settings represented |  |  |  |  |  |
| Equity impacts |  |  |  |  |  |
| Level of strategy specification across studies |  |  |  |  |  |

29. We describe **Study Design Quality** as the quality of the study designs used to evaluate a strategy. We propose this would include the following specific items:

 For Quantitative Studies

- Use of a control arm or comparison group
- Pre/post implementation outcome measurement
- Approaches to bias control (i.e., randomization, sampling)
- Extent of Limitations
- Adequacy of power

For Qualitative Studies

- Rationale for participant selection
- Rationale for data collection approach
- Rationale for data analysis approach
- Description adequacy for data collection, management, and transcription
- Description adequacy for data analysis, codes, and code definitions.
- Use of intercoder reliability techniques.
- Extent of Limitations

For Mixed Method Studies

- Extent to which methods complement or support one another

30. Are there any items you would add?

- No
- Yes

31. If yes, what?

________________________________________________________________

32. Are there any you would drop?

- No
- Yes

33. If yes, what?

________________________________________________________________

34. In your opinion, how important or unimportant are these **Quantitative Study Design** items for the Study Design Quality domain they represent

|  | Very unimportant | Somewhat unimportant | Neither important or unimportant | Somewhat important | Very important |
| --- | --- | --- | --- | --- | --- |
| Use of a control arm or comparison group |  |  |  |  |  |
| Pre/post implementation outcome measurement |  |  |  |  |  |
| Approaches to bias control (i.e., randomization, sampling) |  |  |  |  |  |
| Extent of Limitations |  |  |  |  |  |
| Adequacy of power |  |  |  |  |  |

35. In your opinion, how important or unimportant are these **Qualitative Study Design** items for the Study Design Quality domain they represent

|  | Very unimportant | Somewhat unimportant | Neither important or unimportant | Somewhat important | Very important |
| --- | --- | --- | --- | --- | --- |
| Rationale for participant selection |  |  |  |  |  |
| Rationale for data collection approach |  |  |  |  |  |
| Rationale for data analysis approach |  |  |  |  |  |
| Description adequacy for data collection, management, and transcription |  |  |  |  |  |
| Description adequacy for data analysis, codes, and code definitions |  |  |  |  |  |
| Use of intercoder reliability techniques |  |  |  |  |  |
| Extent of Limitations |  |  |  |  |  |

36. In your opinion, how important or unimportant is this **Mixed Methods Study Design** item for the Study Design Quality domain they represent

|  | Very unimportant | Somewhat unimportant | Neither important or unimportant | Somewhat important | Very important |
| --- | --- | --- | --- | --- | --- |
| Extent to which methods complement or support one another |  |  |  |  |  |

37. We describe **Implementation Outcomes** as the type of implementation outcomes measured, operationalization of the implementation outcomes, and quality of measurement of the implementation outcomes. We propose this would include the following specific items:

 • Operationalization of implementation outcomes
 • Validity and reliability of measures used
 • Quant only: Significance and direction of effect
 • Qual only: Reported effects, either beneficial, neutral, or harmful.

38. Are there any items you would add?

- No
- Yes

39. If yes, what?

________________________________________________________________

40. Are there any you would drop?

- No
- Yes

41. If yes, what?

________________________________________________________________

42. In your opinion, how important or unimportant are these items for the Implementation Outcomes domain they represent

|  | Very unimportant | Somewhat unimportant | Neither important or unimportant | Somewhat important | Very important |
| --- | --- | --- | --- | --- | --- |
| Operationalization of implementation outcomes |  |  |  |  |  |
| Validity and reliability of measures used |  |  |  |  |  |
| Quant only: Significance and direction of effect |  |  |  |  |  |
| Qual only: Reported effects, either beneficial, neutral, or harmful |  |  |  |  |  |

43. We describe the domain **Equity Impact**as the impact of a strategy on promoting health equity. We propose this would include the following specific items:

 • Consideration of equity in research design
 • Effect on equity-related outcomes

44. Are there any items you would add?

- No
- Yes

45. If yes, what?

________________________________________________________________

46. Are there any you would drop?

- No
- Yes

47. If yes, what?

________________________________________________________________

48. In your opinion, how important or unimportant are these items for the Equity Impact domain they represent

|  | Very unimportant | Somewhat unimportant | Neither important or unimportant | Somewhat important | Very important |
| --- | --- | --- | --- | --- | --- |
| Consideration of equity in research design |  |  |  |  |  |
| Effect on equity-related outcomes |  |  |  |  |  |

49. We describe the domain **Strategy Specification**as the rationale for selecting a strategy (i.e., theory, identified barriers, and mechanisms). The quality of specification of a strategy for future practice or research or for replicability of a strategy. We propose this would include the following specific items:

 • Theoretical justification for strategy selection
 • Rationale for how the strategy works and why it should be effective.
 • Level of detail regarding the context in which the strategy was tested
 • Level of detail regarding the barriers and facilitators addressed by the strategy.
 • Level of detail regarding the implementation strategy itself

50. Are there any items you would add?

- No
- Yes

51. If yes, what?

________________________________________________________________

52. Are there any you would drop?

- No
- Yes

53. If yes, what?

________________________________________________________________

54. In your opinion, how important or unimportant are these items for the Strategy Specification domain they represent

|  | Very unimportant | Somewhat unimportant | Neither important or unimportant | Somewhat important | Very important |
| --- | --- | --- | --- | --- | --- |
| Theoretical justification for strategy selection |  |  |  |  |  |
| Rationale for how the strategy works and why it should be effective |  |  |  |  |  |
| Level of detail regarding the context in which the strategy was tested |  |  |  |  |  |
| Level of detail regarding the barriers and facilitators addressed by the strategy |  |  |  |  |  |
| Level of detail regarding the implementation strategy itself |  |  |  |  |  |

55. We describe the domain **Bundled Strategies**as special considerations for bundled strategies (i.e., multiple strategies that are delivered together), such as justification for bundled strategies and ability to discern effects of individual components. We propose this would include the following specific items:

 • Theoretical justification for strategy selection
 • Analyses demonstrating that strategies are more effective when delivered together
 • Analyses demonstrating the relative value of individual strategies tested

56. Are there any items you would add?

- No
- Yes

57. If yes, what?

________________________________________________________________

58. Are there any you would drop?

- No
- Yes

59. If yes, what?

________________________________________________________________

60. In your opinion, how important or unimportant are these items for the Bundled strategies domain they represent

|  | Very unimportant | Somewhat unimportant | Neither important or unimportant | Somewhat important | Very important |
| --- | --- | --- | --- | --- | --- |
| Analyses demonstrating that strategies are more effective when delivered together |  |  |  |  |  |
| Analyses demonstrating the relative value of individual strategies tested |  |  |  |  |  |
| Theoretical justification for delivering strategies together |  |  |  |  |  |

61. Is there anything else about the sub-components of each domain that you would like to share?

________________________________________________________________

________________________________________________________________

________________________________________________________________

Domains of Evidence

62. **Levels of Evidence**

There is also a need for developing levels of evidence strength. We are proposing five evidence levels for implementation strategies: best practice strategies, promising strategies, emerging strategies, undetermined strategies and not recommended strategies. Please read the evidence levels and the associated recommendations. We have included preliminary descriptions of each to provide additional information but plan to revise these descriptions and provide more details based on the results of the Delphi. For now, please consider the evidence levels and recommendations, and respond to the questions below.

| **Evidence Level** | **Recommendation** |
| --- | --- |
| Best Practice Strategy | Strategies demonstrate consistent positive outcomes from high quality research. These strategies would be universally recommended across contexts for the barriers they are intended to address. |
| Promising Strategy | Strategies demonstrate mostly positive outcomes from medium quality research. These strategies may need more rigorous examination to ensure they are having the intended effect or are generalizable to a wider context. Would recommend caution when using them in practice. |
| Emerging Strategies | Strategies demonstrate initial positive outcomes from limited research. These strategies would not be recommended for use in practice without further scientific investigation. These strategies would be recommended for continued research. |
| Undetermined Strategies | Strategies demonstrate mixed outcomes from medium quality research. These strategies would not be recommended for use in practice without further scientific investigation. These strategies would be recommended for continued research using more rigorous research designs. |
| Not Recommended Strategy | Strategies do not demonstrate an effect or demonstrate harmful effects from high quality research. These strategies would not be recommended for the barriers they are intended to address and would not be recommended for further scientific investigation. |

63. Do you feel that there should be other levels?

- No
- Yes

64. If yes, what would it be?

________________________________________________________________

65. Do you feel any of these levels should be dropped?

- No
- Yes

66. If yes, which one(s)?

________________________________________________________________

67. Should any of these levels be combined?

- No
- Yes

68. If yes, which ones?

________________________________________________________________

69. Would you revise the recommendation associated with any of the levels?

- No
- Yes

70. If yes, how would you revise them?

________________________________________________________________

71. Is there anything else about evaluating the science supporting implementation strategies for HIV prevention and treatment services that you would like to share with us?

________________________________________________________________

________________________________________________________________

Delphi Survey Round 2

Introduction

The Implementation Science Coordination Initiative (ISCI) team at Northwestern University promotes high-quality implementation science and creates opportunities to synthesize generalizable knowledge in HIV research and practice funded through the Ending the HIV Epidemic (EHE) Initiative. For those not familiar with implementation science or its application in practice-based settings, implementation science is the systematic study of how to promote adoption and integration of evidence-based practices, interventions, and policies into routine health care and public health settings, to improve the impact on population health.

**Our focus in this survey is on implementation strategies or the things researchers, providers, and others do to help promote intervention implementation.** Examples can include restructuring organizations or clinical care, providing new training to staff, partnering with other agencies, creating incentives for staff or clients, and so forth. Implementation research tests these strategies scientifically to determine whether they work in addressing barriers and supporting implementation. However, we do not have criteria for evaluating quality of science supporting implementation strategies or for making recommendations to practitioners about which strategies are most beneficial. To begin establishing criteria for strategies related to HIV interventions, we conducted a review of the scientific literature, interviewed a range of key informants, and evaluated a preliminary tool in the first round of a Delphi. From the Delphi Round 1, we adjusted some of the domains and evidence levels for the tool based on your responses.

**In this second Delphi round, we need your input on the changes made, and ask you to apply the tool to specific case studies of HIV implementation strategy research to assess its applicability and learn about your experience in applying it.** We will start by asking about your experience with implementation research and your area of expertise (* indicates a required response).

1. I work primarily in (select all that apply)*

- Clinical settings
- Public health settings
- Academic settings
- Federal Government settings
- Other

2. Please Specify.

________________________________________________________________

3. I spend a significant percent of my time (select all that apply)*

- Providing services directly to patients or clients
- Researching interventions, practices, or policies for scientific purposes
- Evaluating interventions, practices, or policies for organizational quality improvement
- Determining and providing funding for health services
- Developing or implementing policies that influence the provision of health services
- Other

4. Please specify

________________________________________________________________

5. I work in the following areas (select all that apply)*

- HIV
- Mental Health
- Substance Misuse
- Social Services
- Primary Care
- Women's Health
- Another area of health

6. Please specify the other area of health

________________________________________________________________

7. How would you describe your level of knowledge of implementation science (e.g., theories, frameworks, implementation outcomes)?*

- I know almost nothing about implementation science
- I know about the basic components of implementation science
- I know a moderate amount about implementation science
- I know a lot about implementation science

8. What would you say is your level of experience in implementation research? (Select all that apply)*

- I have not formally been involved in implementation research
- I have supported implementation research projects
- I have led implementation research projects
- I have been a partner in implementation research

9. How many years of experience do you have in your field?

- 0-10 years
- 11-20 years
- 21-30 years
- 31 years or more

10. What is your race? (Select all that apply)

- American Indian or Alaska Native
- Asian
- Black or African American
- Native Hawaiian or other Pacific Islander
- White
- Other __________________________________________________
- Prefer not to respond

11. What is your ethnicity?

- Hispanic or Latino
- Not Hispanic or Latino

12. How do you describe yourself? (Select all that apply)

- A man
- A woman
- Nonbinary
- Prefer not to respond

Introduction to Tool and Domains

Instructions:
We would like you to begin by reviewing the tool, the criteria domains used to evaluate the evidence and the evidence levels. We will then get your feedback on some of the changes that were made to the tool in response to the Delphi Round 1 results. In case you would like to look at the full-length descriptions of both old and new versions of the tool while answering the questions, we have provided the descriptions as a pdf you can select and open in a separate window indicated by the X symbol. 

Overview of the tool
This tool is intended to provide criteria to evaluate the scientific evidence supporting implementation strategies, **specifically relevant to HIV interventions**. This tool is intended to help differentiate among implementation strategies within the existing scientific HIV implementation science literature. This will allows us to make recommendations to the practice and research community about HIV implementation strategies with the best evidence. 

While this means that it will differentiate evidence quality supporting the effectiveness of implementation strategies for HIV, it would not be directly applicable to other areas of implementation science where there may be a more or less robust evidence base.

Criteria Domains
The criteria for the tool used to evaluate the evidence for HIV-related implementation strategies are divided into five domains - study design, implementation outcomes, study rigor and limitations, strategy specification, and equity. All documentation related to a strategy that can inform the various domains is considered, for instance, pre-implementation barrier/facilitator assessments, protocol papers, and reported outcomes. Taken together, we consider the overall body of scientific evidence supporting strategy quality across each of these domains (i.e., overall effectiveness). The domains are described as follows:

**Study design –** The elements of study design(s) used to evaluate a strategy, primarily, via the use of a comparison group and assessment before (pre) and after (post) a strategy is used.

**Implementation outcomes –** The effect and effect direction of deliberate and purposive actions to implement new treatments, practices, and services. Implementation outcomes include knowledge or awareness, appropriateness, acceptability, feasibility, adoption or uptake, fidelity or adherence, cost, sustainment, penetration, or reach. We are taking an expanded definition of implementation outcomes to include antecedent/proximal outcomes (e.g., knowledge, awareness) and patient use/adherence.

**Study rigor and limitations –** Limitations that may influence the validity or generalizability of the results as appropriate for different study designs including quantitative, qualitative and mixed-method designs.

**Strategy Specification –** The level of specificity about the strategy for purposes of reproducibility. More specifically, whether the rationale for strategy selection and connection to the barriers they are intended to impact are given; whether description using recommendations for specifying an implementation strategy is adequate; and whether description of the context where the strategy is deployed is provided. If multi-strategy or a blended strategy approach is used, whether a rationale for the approach is provided.

**Equity Impact –** The impact of a strategy among target populations disproportionally affected by the HIV epidemic as identified by the Center for Disease Control and Prevention, distinguishing between strategies that examine disparities and those that promote health equity. We define disparities research as research that examines differences among target populations. We define health equity research as research that intentionally promotes better equity and reduces disparities in health outcomes for target populations. Target populations include men who have sex with men (e.g., gay and bisexual men), African Americans, Latinx individuals, people who inject drugs, or transgender individuals. This domain also considers the extent to which target populations have informed strategy development and research design.

In general, respondents wanted greater specificity for the study design domain and criteria within the domain. In response to this critique, we split this domain into two separate smaller domains and criteria within each domain. The original domain, "Study Design Quality" was split into "Study Design" and "Study Rigor and Limitations". The original and new domains are described in the table below. 

| **Original Domain** | **New Domains** | |
| --- | --- | --- |
| Study Design Quality | Study Design | Study Rigor and Limitations |
| The quality of the study designs used to evaluate a strategy. | The elements of study design(s) used to evaluate a strategy, primarily, via the use of a comparison group and assessment before (pre) and after (post) a strategy is used. | Limitations that may influence the validity or generalizability of the results as appropriate for different study designs including quantitative, qualitative and mixed-method designs. |

13. Do you agree with this change?

- Yes
- No

15. If no, please explain why.

________________________________________________________________

________________________________________________________________

Respondents felt that a domain dedicated to bundled or multi-component strategies was not necessary. Based on the Delphi Round 1 results, we removed the "Bundled Strategies Domain". We moved the relevant criteria for bundled strategies into the "Strategy Specification" domain as suggested by respondents. Both the old bundled strategies and new strategy

specification domain descriptions are in the table below. 

| **Original Domain** | **New Domain** |
| --- | --- |
| Bundled Strategies | Strategy Specification |
| Special considerations for bundled strategies (i.e., multiple strategies that are delivered together), such as justification for bundled strategies and ability to discern effects of individual components." | The level of specificity about the strategy for purposes of reproducibility. More specifically, whether the rationale for strategy selection and connection to the barriers they are intended to impact are given; whether description using recommendations for specifying an implementation strategy is adequate; and whether description of the context where the strategy is deployed is provided. If multi-strategy or a blended strategy approach is used, whether a rationale for the approach is provided. |

16. Do you agree with this change?

- Yes
- No

17. If no, please explain why.

________________________________________________________________

________________________________________________________________

There was some divergence in opinion about the proposed equity domain with some suggesting that equity could be included as an implementation outcome and some felt like it should be more specific and comprehensive. We have opted to keep the domain, but provide more clarity and specificity about the domain including target populations, whether communities are engaged in the research, and whether the intent of the research is to promote equity or reduce disparities in addition to the impact on outcomes. The original and revised descriptions are provided in the table below. 

| **Original Domain** | **New Domain** |
| --- | --- |
| Equity | Equity Impact |
| - The impact of a strategy on promoting healthy equity. | - The impact of a strategy among target populations disproportionally affected by the HIV epidemic as identified by the Center for Disease Control and Prevention, distinguishing between strategies that examine disparities and those that promote health equity. We define disparities research as research that examines differences among target populations. We define health equity research as research that intentionally promotes better equity and reduces disparities in health outcomes for target populations. Target populations include men who have sex with men (e.g., gay and bisexual men), African Americans, Latinx individuals, people who inject drugs, or transgender individuals. This domain also considers the extent to which target populations have informed strategy development and research design. |

18. Do you agree with keeping this domain separate?

- Yes
- No

19. If no, please explain why.

________________________________________________________________

Levels of Evidence 
We have defined four possible evidence levels for HIV-related implementation strategies: best practice, promising practice, needing more evidence, and non-recommended strategies. These levels are intended to help distinguish between implementation strategies that are likely to improve implementation outcomes for HIV prevention and treatment interventions equitably among populations disproportionally impacted by HIV, and those which require more research to demonstrate positive and equitable impact. The descriptions of minimum requirements for each of levels is as follows: 

**Best Practice Strategies –** Strategies would be recommended for uptake in practice to address barriers in contexts where studied. To meet this level, strategies must demonstrate effectiveness on primary implementation outcomes. Evidence is generated from at least one well-designed study that has at a minimum a pre/post assessment or a comparison group with minimal limitations. Adequate strategy specification is available to sufficiently replicate and/or adapt the strategy. Health equity is improved for populations who experience inequitable outcomes related to HIV.

**Promising Strategies –** Strategies may be used for uptake in practice to address barriers in contexts where trialed, but users should carefully monitor implementation outcomes to ensure strategies are having intended effect. To meet this level, strategies must demonstrated effectiveness on primary implementation outcomes, but validity or generalizability of results is more limited. Evidence is generated from at least one study with a pre/post assessment or a comparison group. Some strategy specification is available to replicate and/or adapt the strategy, but some inference may be needed. Health equity may go unaffected for populations who experience inequitable outcomes related to HIV. More rigorous examination of these strategies would be ideal to ensure they are having the intended effect.

**More Evidence Needed –** Additional scientific examination of these strategies is needed before recommendation for use in practice setting. To meet this level, strategies demonstrate effectiveness in a pilot or feasibility study. Studies for which the main effects are null and simulation studies examining strategies are also considered in need of more evidence. Studies may have also major limitations that limit validity or generalizability of the results, and/or the strategy is poorly specified. Health equity is unexamined for populations who experience inequitable outcomes related to HIV.

**Non-Recommended Strategies –** Strategies would not be recommended for the barriers they are intended to address. Studies identify harmful or worsening effects on implementation outcomes or outcomes that potentially exacerbate disparities among target populations.

Originally there were five evidence levels in our tool, but participants felt that there were too many evidence levels and indicated it was difficult to distinguish among them. Based on Delphi Round 1 results, we combined the "Emerging" and "Undetermined" strategy levels which is now described as "More Evidence Needed" and revised the level definitions to create greater clarity and distinction between levels. All other domain levels including best practice, promising practice, and non-recommended strategies have remained the same with minimal revisions to the description. 

| **Original Evidence Levels** | | **New Evidence Level** |
| --- | --- | --- |
| Emerging Strategy | Undetermined Strategy | More Evidence Needed |
| Strategies demonstrate initial positive outcomes from limited research. These strategies would not be recommended for use in practice without further scientific investigation. These strategies would be recommended for continued research. | Strategies demonstrate mixed outcomes from medium quality research. These strategies would not be recommended for use in practice without further scientific investigation. These strategies would be recommended for continued research using more rigorous research designs. | Additional scientific examination of these strategies is needed before recommendation for use in practice setting. To meet this level, strategies demonstrate effectiveness in a pilot or feasibility study. Studies for which the main effects are null and simulation studies examining strategies are also considered in need of more evidence. Studies may have also major limitations that limit validity or generalizability of the results, and/or the strategy is poorly specified. Health equity is unexamined for populations who experience inequitable outcomes related to HIV. |

20. Do you agree with the number of evidence levels?

- Yes
- No

21. If no, please explain why.

________________________________________________________________

22. In the Delphi Round 1, you were asked to rank the domains in order of importance. The results ranked the domains in the following order:
 1. study design
 2. implementation outcomes
 3. strategy specification
 4. equity

23. In alignment with these results, we have ordered the domains hierarchically so that the evidence for strategies is considered in the same order with study rigor and limitations added. This indirectly gives more weight to earlier domains. For instance, an implementation study would automatically be assigned to the evidence level "More evidence needed" regardless of performance on subsequent domains if the primary implementation outcome is null. 

 Do you agree with the order of the domains?

- Yes
- No

24. If no, please explain why.

________________________________________________________________

25. Do you have other suggestions for ordering the domains?
