# Supplementary material for: Establishing evidence criteria for implementation strategies in the US: a Delphi study for HIV services
Source: Implement Sci. 2024 Jul 15;19:50. doi: 10.1186/s13012-024-01379-3 (PMC11251241; doi:10.1186/s13012-024-01379-3)
Supplement: Supplementary file 3 — Supplementary Material 3. [file 13012_2024_1379_MOESM3_ESM.docx]

**Best Practice Strategies Evidence Tool**

**Overview of the tool**

This tool is intended to provide criteria to evaluate the scientific evidence supporting implementation strategies. We define implementation strategies as methods or techniques to improve the adoption, implementation, and sustainability of an intervention.^[[1]](#footnote-2)^ The criteria are divided into five domains including: research design, implementation outcomes, study rigor and limitations, strategy specification, and equity. Taken together, we consider the overall body of scientific evidence supporting strategy quality across each of these domains (i.e., overall effectiveness). We have defined four possible evidence levels: best, promising, emerging, and non-recommended strategies. These levels are intended to help distinguish between implementation strategies that are recommended for practices that are likely to improve implementation outcomes for HIV prevention and treatment interventions equitably among populations impacted by HIV, and those which require more research to demonstrate positive and equitable impact.

**Tool Development**

This tool was developed iteratively beginning with a review of the literature and existing rubrics used to evaluate scientific evidence for health intervention.^[[2]](#footnote-3)^ We conducted key informant interviews to understand the extent to which experts felt existing rubrics and evaluation criteria apply to implementation strategies. Based on these interviews, we developed an initial tool and set of criteria. We then invited investigators and practitioners from a variety of fields and levels of experience with implementation science to give their opinion on and revise these criteria. Members of the ISCI team then reviewed the revised rubric and applied it to existing evidence supporting implementation strategies designed to promote the uptake of evidence-based intervention for HIV prevention and treatment.

**Criteria Domains**

The criteria for the tool are divided into five domains - study design, implementation outcomes, study rigor and limitations, strategy specification, and equity. All documentation related to a strategy that can inform the various domains is considered, for instance, pre-implementation barrier/facilitator assessments, protocol papers, and reported outcomes. The domains are described as follows and more specific criteria included in each of the domains is provided in the tables below.

Study design –The elements of study design(s) used to evaluate a strategy primarily the use of a comparison group and assessment before (pre) and after (post) a strategy is used.

Implementation outcomes –The effect and effect direction of deliberate and purposive actions to implement new treatments, practices, and services.^[[3]](#footnote-4)^ Implementation outcomes include knowledge or awareness, appropriateness, acceptability, feasibility, adoption or uptake, fidelity or adherence, cost, sustainment, penetration, or reach.

Study rigor and limitations –Limitations that may limit the internal validity or generalizability of the results.

Strategy Specification – The level of specificity about the strategy for purposes of reproducibility. More specifically, whether the rationale for strateg(ies) selection and connection to the barriers they are intended to impact are given; whether strategy description using recommendations for specifying an implementation strategy is adequate;^[[4]](#footnote-5)^ and whether description of the context where the strategy is deployed is provided. If multi-strategy or a blended strategy approach is used, whether a rationale for the approach is provided.

Equity Impact – The impact of a strategy among target populations disproportionally affected by the HIV epidemic.^[[5]](#footnote-6)^. We define health equity research as research that intentionally promotes better equity and reduces disparities in health outcomes for target populations. Target populations experiencing inequities as defined by the Centers for Disease Control and Prevention include men who have sex with men (e.g., gay and bisexual men), African Americans, Latinx individuals, people who inject drugs, or transgender individuals. However, we also consider other populations for which disparities and inequities are well defined in the scientific literature. This domain also considers the extent to which target populations have informed strategy development and research design.^[[6]](#footnote-7)^

**Levels of Evidence**

There are four levels of evidence supporting the strategies, based on their generalizability across contexts, whether it is recommended for use among practitioners or whether more research is needed for the strategy. The descriptions of minimum requirements for each of the levels is as follows:

Best Practice Strategies – Strategies that would be recommended for uptake in practice to address barriers in contexts where studied. To meet this level, strategies must demonstrate positive effectiveness on primary implementation outcomes. Evidence is generated from at least one well-designed study that has at a minimum a pre/post assessment or a comparison group with minimal limitations. Adequate strategy specification is available to sufficiently replicate and/or adapt the strategy. Health equity is improved for populations who experience inequitable outcomes related to HIV.

Promising Strategies – Strategies may be used for uptake in practice to address barriers in contexts where studied, but users should carefully monitor implementation outcomes to ensure strategies are having intended effect. To meet this level, strategies must demonstrate at least some evidence of effectiveness on primary implementation outcomes, but validity or generalizability of the results is more limited. Evidence is generated from least one study with a pre/post assessment or a comparison group. Some strategy specification is available to replicate and/or adapt the strategy, but some inference may be needed. Health equity may go unaffected for populations who experience inequitable outcomes related to HIV. More rigorous examination of these strategies would be ideal to ensure they are having the intended effect.

More Evidence Needed – Additional scientific examination of these strategies is needed before recommendation for use in practice settings. This includes preliminary/pilot studies, studies for which the main effects are null, simulation studies, studies with major limitations that limit validity of the results, and/or studies where the strategy is poorly specified. This also includes studies where health equity is unexamined for populations who experience inequitable outcomes related to HIV.

Non-Recommended Strategies – Strategies would not be recommended for the barriers they are intended to address. Studies identify harmful effects or outcomes that potentially exacerbate inequities among target populations.

# Overall Effectiveness by Evidence Level

The table below summarizes the minimum domain criteria required to assign an evidence level. More detailed descriptions are provided in subsequent tables.

| **Best Practice Strategies** | **Promising Strategies** | **More Evidence Needed** | **Non-Recommended Strategies** |
| --- | --- | --- | --- |
| - At least one study with a pre/post assessment *or* a comparison group. - Positive primary implementation outcome effect. - No major limitations to study rigor. - Strategy well specified. - Equity impact demonstrated. | - At least one study with a pre/post assessment *or* a comparison group. - Positive primary implementation outcome effect. - Some limitations to study rigor. - Strategy mostly well specified. - May not improve but does not exacerbate inequities. | - Pilot or feasibility, post-assessment only with no comparison group, or simulation study. - Null primary implementation outcome effect. - Some limitations to study rigor. - Strategy is not well specified - Equity impact not demonstrated or considered. | - Harmful implementation outcome effects reported. |

**How to use this tool**

- The individual criteria for each evidence level and each domain is provided in the tables below.
- At the top of each domain table is a set of instructions on how to apply the criteria for that domain.
- A strategy must meet all of the criteria within the Best Practice or Promising evidence level to meet that level of evidence. Otherwise, the next highest evidence level (to the right) must be considered.
- Assign the evidence level of the strategy for each domain.
- The lowest evidence level assigned among all the domains is the overall rating for the strategy.

# Study Design

| **Instructions:** All criteria within the Best Practice evidence level must be met to be assigned that evidence level otherwise it should be assigned as More Evidence Needed. All studies should be rated as either Best Practice or More Evidence Needed. | | | |
| --- | --- | --- | --- |
| **Best Practice Strategies** | **Promising Strategies** | **More Evidence Needed** | **Non-Recommended Strategies** |
| - Pre/post assessment of implementation outcomes **OR**   has a comparison group  *Example Designs: Multi-site Hybrid II or III trials, natural experiments, within-site repeated measures designs |  | - Identifies as a feasibility or pilot study - Post evaluation only with no comparison group - Simulation Studies   *Example Designs: Pilot studies, post assessment of implementation of strategy only, hybrid type I studies. |  |

# Implementation Outcomes and Effects

| **Instructions:** Consider the primary implementation outcome from the study. If no primary outcome is identified in the study, consider the most proximal implementation outcome to the strategy. Results related to implementation outcomes from qualitative studies can be considered. All appropriate criteria for the Best Practice evidence level must be met to be assigned that evidence level. If any outcome is harmful, it should be assigned as Non-recommended. Otherwise, it should be assigned as More Evidence Needed. | | | |
| --- | --- | --- | --- |
| **Best Practice Strategies** | **Promising Strategies** | **More Evidence Needed** | **Non-Recommended Strategies** |
| - Primary outcome is operationalized, targeted implementation outcome(s) (e.g. adoption, feasibility) - Primary effect is reported as positive or beneficial. Studies with post only measurement, if the outcome is accomplished, then the outcome can be considered positive. - Quant studies or mixed methods: Has a significant positive effect on at least one targeted implementation outcome (p<.05) |  | - Implementation outcome(s) (e.g. adoption, feasibility) are mentioned but perhaps not operationalized - Primary effect from primary study is null or not achieved as intended. - Quant studies or mixed-method: Has positive effect, but at less stringent significant criteria (p<.1) | - Demonstrates harmful implementation outcomes (primary or secondary) |

# Study Rigor and Limitations

**Instructions:** Consider the additional design criteria of the study to evaluate for major limitations to validity or generalizability of the research. For qualitative studies, criteria only need be described (no determination of adequacy or quality necessary). For mixed method studies with a primary method (i.e., primarily quantitative or primarily qualitative), criteria need only be met for the primary method. For mixed method studies with balanced methods (i.e., quantitative and qualitative methods are equal), criteria for both methods must be met. In addition, methods in mixed method studies must complement each other.

For the appropriate study design begin at the Best Practice evidence level. All criteria must be met at that level, otherwise consider the Promising evidence level. If the study does not meet all criteria at the Promising evidence level, then it is assigned as Needs More Evidence.

| **Best Practice Strategies** | **Promising Strategies** | **More Evidence Needed** | **Non-Recommended Strategies** |
| --- | --- | --- | --- |
| Quant studies:   - Approaches to bias minimization used (e.g., randomization, matching, statistical comparison of intervention and control sampling, sample weighting, etc.) - Study includes multiple sites or replication has occurred.     Qual studies:   - Adequate description of data collection procedures, management, and transcription - Adequate description of analysis, analytic codes and code definitions - Intercoder reliability or member checking conducted during analyses if multiple coders are used     Mixed Method Studies:   - Methods complement one another | Quant studies:   - Approaches to bias minimization used (e.g., randomization, matching, statistical comparison of intervention and control samples, sample weighting, etc.) - Study conducted at a single site with no replication.     Qual studies:   - Adequate description of data collection procedures, management, and transcription - Adequate description of analysis, analytic codes and code definitions - Intercoder reliability or member checking conducted during analyses if multiple coders are used     Mixed Method Studies:   - Methods complement one another | Quant studies:   - Approaches to bias minimization not used (e.g., randomization, statistical comparison of intervention and control samples, sample weighting, etc.) - Study conducted at a single site with no replication     Qual studies:   - Inadequate description of multiple aspects of the research design including data collection approach and analysis.     Mixed Method Studies:   - Unclear whether methods complement one another |  |

# Strategy Specification for Future Implementation and Replication

| **Instructions:** For all available materials about a strategy, evaluate quality of strategy specification, context description, and connection to identified determinants. While individual manuscripts need not meet all criteria, collectively all items must be addressed for a strategy. The reviewer may infer elements of strategy specification if they are not described using these terms overtly. For the appropriate strategy design begin at the Best Practice evidence level. If all appropriate criteria are met at that level, the reviewer can stop and assign the rating. Otherwise consider the criteria for the Promising evidence level. If the study meets all the criteria at the Promising evidence level, then the reviewer can stop and assign the rating. Otherwise assign the evidence level Needs More Evidence.  For strategy specification Proctor’s recommendations for implementation strategy operationalization should be used defined as:  Actor – Identify who enacts the strategy. Who is responsible for enacting the strategy?  Action – The specification actions, steps, or processes that need to be enacted. In a general sense, what does the strategy entail? While it’s not expected that the authors provide every detail, the reader should be able to consider whether the strategy could potentially be used in their context. Ideally, they would do more reading to make a final determination and get more details.  Target – Unit of analysis for measuring implementation outcomes. What is expected to change because of the strategy? All included strategies in this review should meet this criteria.  Temporality – Timing or sequencing of strategy. When should the strategy be used? For this element, the review can assume that the strategy would naturally be employed immediately or at any time if appropriate. However, some strategies may require specific timing, e.g., getting collaborative agreements in place before implementing a referral program. In these cases, it should be described in the paper.  Dose – Dose or intensity of strategy including frequency or length of time.  How often should the strategy be used? Similar to above, the reviewer can assume the strategy should naturally be used all the time or always if appropriate. However, some strategies may involve a specific dose, e.g., educational programs. In these cases, it should be described in the paper. | | | |
| --- | --- | --- | --- |
| **Best Practice Strategies** | **Promising Strategies** | **More Evidence Needed** | **Non-Recommended Strategies** |
| - Context: Studies detail the context(s) in which the strategy was implemented including type(s) of agencies, demographics of participants at the deliverer and recipient-levels. - Barriers/Facilitators: Studies discuss the potential barriers and facilitators to implementation - Strategy selection Rationale: There is a rationale for how the strategy works or why it should be effective (i.e., theoretical or empirical connection to barrier(s)) - Strategy elements: All strategy elements are mentioned or can be inferred as appropriate - For blended or multi-component strategies: Justification for the blending of strategies or utilization | - Context: Studies detail the context(s) in which the strategy was implemented including type(s) of agencies, demographics of participants at the deliverer and recipient-levels. - Barriers/Facilitators: Studies discuss potential barriers and facilitators to implementation - Unclear rationale for how the strategy works or why it should be effective. - Strategy elements: Majority (3 of 5) of strategy elements are mentioned or can be inferred as appropriate but some aspects may be insufficiently specified. - For blended or multi-component strategies: Lacks justification for the blending of strategies or utilization | - Context: Studies do not detail the context(s) in which the strategy was implemented including type(s) of agencies, demographics of participants at the deliverer and recipient-levels. - Barriers/facilitators: Studies do not discuss potential barriers and facilitators to implementation - Unclear rationale for how the strategy works or why it should be effective. - Majority of strategy elements are not specified. - For blended or multi-component strategies: lacks justification for the blending of strategies or utilization |  |

# Equity Impact

| **Instructions:** For all available materials about a strategy, examine the equity impact of the strategy based on the communities targeted in the research, the outcomes for these groups at either the level of implementation or health outcomes, and their engagement in the research process. As a reminder, target populations for HIV prevention and treatment include but are not limited to men who have sex with men (e.g., gay and bisexual men), African Americans, Latinx individuals, people who inject drugs, or transgender individuals. Other populations may be considered if an inequity has been identified. While individual manuscripts need not meet all criteria, collectively all criteria must be addressed within an evidence level to maintain the evidence level assigned. First check to make sure there are no negative effects that would increase inequities. If yes, then assign as non-recommended and stop. Then begin at the Best Practice evidence level for the appropriate strategy design. If all appropriate criteria are met at that level, the reviewer can stop and assign the rating. Otherwise consider the criteria for the Promising evidence level. If the study meets all the criteria at the Promising evidence level, then the reviewer can stop and assign the rating. Otherwise assign the evidence level Needs More Evidence. | | | |
| --- | --- | --- | --- |
| **Best Practice Strategies** | **Promising Strategies** | **More Evidence Needed** | **Non-Recommended Strategies** |
| - Includes target populations experiencing inequities - Has a positive effect on target populations that improves equity or reduces disparities specifically on a target population - Frames research in terms of equity promotion (e.g., describes inequity in introduction or proposes aims to reduce inequities or is informed by an equity framework) - Uses formative research with community, directly engages communities during the study, OR strategy improves interaction with community/patients. | - Includes target populations experiencing inequities - Examines differences in outcomes based on target populations. - Has positive outcomes but does not necessarily improve equity | - Does not focus on health equity - Excludes populations experiencing inequity without rationale | - Found to have a negative effect on health equity (*i.e.,* increases disparities) |

1. Proctor EK, Powell BJ, McMillen JC. Implementation strategies: recommendations for specifying and reporting. Implement Sci. 2013;8:1–11 [↑](#footnote-ref-2)
2. Built on similar work by Smith et al. (2020), the Centers for Disease Control & Prevention’s Compendium of Evidence-Based and Evidence Informed Interventions for HIV Preventions (2016), Pinnock et al. (2016), and Guyatt et al. (2008). Promising strategies category was heavily influenced by Psihopaidas et al. (2020). [↑](#footnote-ref-3)
3. Proctor E, Silmere H, Raghavan R, Hovmand P, Aarons G, Bunger A, Griffey R, Hensley M. Outcomes for implementation research: conceptual distinctions, measurement challenges, and research agenda. Administration and policy in mental health and mental health services research. 2011 Mar;38(2):65-76. [↑](#footnote-ref-4)
4. Proctor EK, Powell BJ, McMillen JC. Implementation strategies: recommendations for specifying and reporting. Implementation Science. 2013 Dec;8(1):1-1. [↑](#footnote-ref-5)
5. Centers for Disease Control and Prevention. Populations at Greatest Risk. <https://www.cdc.gov/hiv/policies/hip/risk.html>. Accessed January 1, 2023. [↑](#footnote-ref-6)
6. Centers for Disease Control and Prevention. Defining and measuring disparities, inequities, and inequalities in the Healthy People Initiative. <https://www.cdc.gov/nchs/ppt/nchs2010/41_klein.pdf> Accessed January 1, 2023. [↑](#footnote-ref-7)
